# Preventive Cardiovascular Measures in Children with Elevated Blood Pressure

**DOI:** 10.3390/life14081001

**Published:** 2024-08-12

**Authors:** Mirjam Močnik, Nataša Marčun Varda

**Affiliations:** 1 Department of Paediatrics, University Medical Centre Maribor, Ljubljanska Ulica 5, 2000 Maribor, Slovenia; 2 Medical Faculty, University of Maribor, Taborska 8, 2000 Maribor, Slovenia

**Keywords:** hypertension, children, prevention, cardiovascular risk

## Abstract

Cardiovascular diseases are the leading cause of morbidity and mortality in developed countries and are becoming increasingly significant in developing nations. Many cardiovascular risk factors originate early in life, even prenatally. Elevated blood pressure and hypertension are gaining attention in paediatrics due to their rising prevalence and impact on early cardiovascular risk in adulthood. Along with non-modifiable risk factors for hypertension, several modifiable factors can be addressed through primordial and primary prevention, emphasising lifestyle modifications. When these measures fail and elevated blood pressure develops, early detection is crucial (secondary prevention). Regular blood pressure measurement, a simple and non-invasive procedure, should be a standard practice in paediatric clinical settings. Diagnosing elevated blood pressure and hypertension should adhere to recommended guidelines. Lifestyle modifications are the first-line therapy for primary hypertension in children; if a secondary cause is identified, targeted treatment can be implemented, but a healthy lifestyle remains essential. The early detection and treatment of high-risk blood pressure enable timely interventions to prevent complications (tertiary prevention). Collectively, these preventive measures aim to reduce the incidence of cardiovascular disease in adulthood. Furthermore, quaternary prevention seeks to avoid unnecessary or harmful medical interventions including unwarranted examinations and pharmacotherapy. This underscores the importance of accurate diagnosis and treatment of elevated blood pressure and hypertension and emphasises the need for primordial and primary prevention to minimise unnecessary clinical interventions.

## 1. Introduction

Globally, ischaemic heart disease and stroke, the most common cardiovascular diseases (CVDs), predominate as leading causes of age-standardised deaths [1]. Despite long-standing gains in life expectancy [1], CVDs could be significantly reduced even further, providing reduced morbidity and mortality from atherosclerosis-associated CVDs and increasing the quality of life by promoting healthy lifestyle practices and preventive measures. These should commence already in childhood, since growing evidence in the young shows increasing or stagnating trends in incident with increasing unhealthy risk factors including obesity, poor diet, and physical inactivity [2], also leading to the increased prevalence of hypertension in childhood [2].

This review emphasises the importance of blood pressure (BP) monitoring and prevention in children as increasing hypertension in the paediatric population contributes to several CVDs and is associated with other cardiovascular (CV) factors.

## 2. Elevated Blood Pressure in Children

Elevated BP and hypertension are increasing in the paediatric population. Current recommendations released by the European Society of Hypertension [3], American Academy of Pediatrics [4], and Hypertension Canada’s 2020 guidelines [5] have identified four categories of BP readings:

– Normal BP;
– Elevated BP (not defined in Hypertension Canada’s 2020 guidelines);
– Stage 1 hypertension;
– Stage 2 hypertension.

Definitions of hypertension based on both guidelines are presented in Table 1 [3,4,5]. In our population, elevated BP was estimated at approximately 7.1% [6], while the incidence of hypertension was estimated to be around 3.5% globally [7]. Paediatric hypertension is diagnosed when a child has three elevated systolic or diastolic BP readings that are above the normal range for their age, height, and sex.

It is important to note, that the 2017 American Academy of Pediatrics revised the normative tables for childhood BP values and excluded overweight and obese children, resulting in an overall increase in the prevalence of hypertension, especially in the young with overweight or obesity [8].

The standard method for confirming a diagnosis of hypertension in children is continuous ambulatory blood pressure monitoring (ABPM) [8,9]. ABPM is particularly useful when white coat hypertension is suspected. It is also recommended for children with high-risk conditions and when there are concerns about poor BP control [8].

Additionally, hypertension can be classified as primary (essential), where no identifiable underlying cause is present, or secondary, where an underlying organic cause is identified. The latter is now less common and characteristic of younger children with severe hypertension. Aetiology ranges from renal, cardiac, endocrine, vascular causes, malignancy, and medications, among others [7]. Primary hypertension is increasing in prevalence in children [7,10], influenced by obesity, genetics, diet, sex, and academic stress, among other associated factors [10].

## 3. Cardiovascular Risk Factors in Childhood

**Figure 1:**
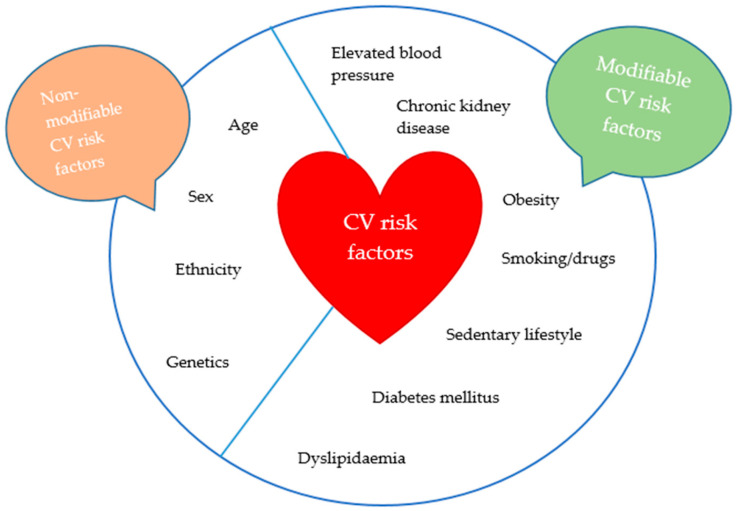
Modifiable and non-modifiable cardiovascular (CV) risk factors.

Hypertension in children is believed to be associated with accelerated atherosclerosis [11]. Even more, while death and cardiovascular disability are rare in hypertensive children, intermediate markers of target organ damage can be detected. These include left ventricular hypertrophy, thickening of the carotid vessel wall, retinal vascular changes, and even subtle cognitive changes in children and adolescents with high BP [12]. Target organ damage is even more pronounced when other/multiple CV risk factors are present [12]. CV factors are divided into modifiable or non-modifiable, as presented in Figure 1. The latter include genetic predisposition, age, sex, and race.

Genetic factors and family history should not be overlooked. Gene polymorphisms of the renin-angiotensin-aldosterone system (RAS), aldosterone synthase gene (CYP11B2), ATPase plasma membrane Ca^2+^ transporting 1 gene (ATP2B1), and endothelial nitric oxide gene polymorphisms have been studied and associated with an increased risk for hypertension [10]. Additionally, the prevalence of hypertension is significantly higher in individuals with a positive family history of the condition [13], suggesting an increased risk due to shared genetic material. However, living in the same environment can also contribute to the development of cardiovascular factors such as obesity due to unhealthy nutrition, which can further lead to obesity-related hypertension, irrespective of genetic factors. In terms of sex, some studies have found an increased risk for essential hypertension in boys [10,14], however, not all studies have confirmed this [15]. Some racial-ethnic differences have been identified, most notably a higher nocturnal BP and abnormal dipping in the Black ethnic group [16], with an encouragement to shift away from simply observing racial disparities. The root causes of these disparities in terms of race—whether physiological, genetic, psychosocial, economic, environmental, governmental, or institutional—must be identified to effectively address and rectify them [17].

On the other hand, several modifiable factors influence CV health, as seen in Figure 1. Typically, these CV risk factors frequently coexist and potentiate the development of each other. For example, obesity and unhealthy diet/sedentary lifestyle are a common denominator for elevated BP, chronic kidney disease, dyslipidaemia, and diabetes mellitus [18]. Recent evidence indicates that hyperuricemia is a significant condition in children and adolescents, especially when linked to noncommunicable diseases. In otherwise healthy young individuals, obesity is a primary cause of hyperuricemia. This condition often coexists with metabolic syndrome; in obese children and adolescents, hyperuricemia is connected to components of metabolic syndrome and noncommunicable diseases such as hypertension, insulin resistance, dyslipidaemia, and chronic kidney disease [19]. Therefore, influencing one CV factor can significantly reduce other CV factors and the overall CV risk. When one risk factor is identified, it is important to search for others and evaluate target organ damage. Consequently, all children with hypertension should be assessed for additional CV risk factors. To minimise CV risk, it is crucial to prevent elevated BP and hypertension in children, along with other CV risk factors or to treat them in a timely manner.

## 4. Preventive Cardiovascular Measures in Childhood Hypertension

To achieve the maximal possible effect in minimising hypertension and subsequent CVDs in children and young adulthood, preventive measures should be aimed at all levels, as presented in Table 2 [20], which also target other associated CV risk factors.

## 5. Primordial and Primary Prevention

**Figure 2:**
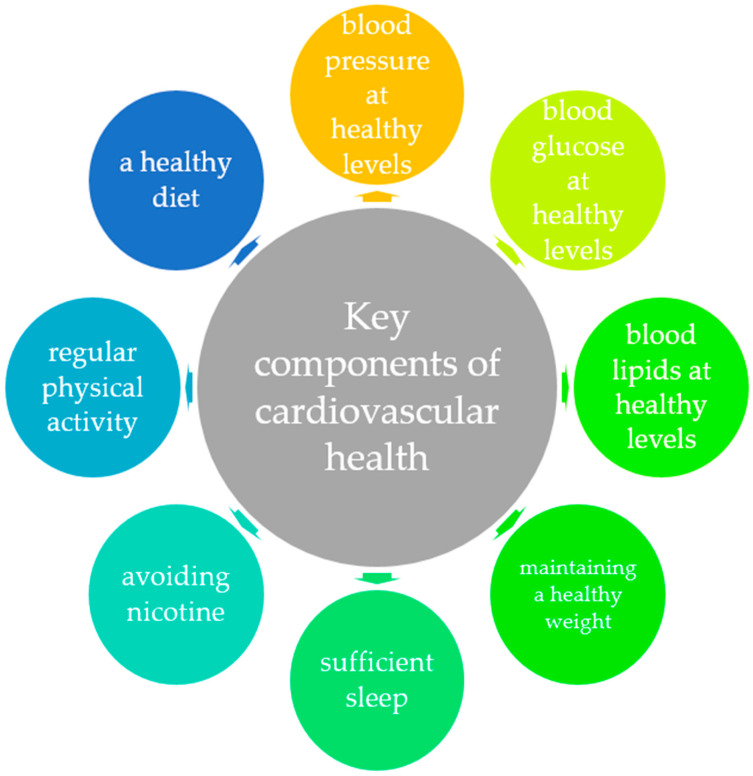
Key components of cardiovascular health.

The primordial prevention of elevated BP and subsequent CVD involves the prevention of the onset of its risk factors [21]. Primordial prevention differs from primary prevention as it focuses on avoiding the development of risk factors altogether, whereas primary prevention involves treating existing risk factors to prevent hypertension development [22]. The American Heart Association (AHA) has identified these risk factors in the adult population under the collective term “Life’s Essential 8”, which encompasses eight key components of CV health: a healthy diet, regular physical activity, avoiding nicotine, sufficient sleep, maintaining a healthy weight, and keeping blood lipids, blood glucose, and blood pressure at healthy levels [23], which all apply to the paediatric population (Figure 2).

While studies consistently identify age as the most significant non-modifiable risk factor for atherosclerotic disease, adopting lifestyle factors that contribute to ideal CV health early on can provide the most protective benefits [21]. Furthermore, events occurring in the early stages of life can significantly influence future CV risk. Therefore, prevention strategies for a child may begin even before pregnancy including measures to ensure the best maternal environment for the foetus through antenatal and perinatal prevention [24]. This is strongly supported by evidence that intrauterine growth restriction and preterm birth are independent risk factors for developing hypertension later in life [25]. Furthermore, maternal hypercholesterolemia, hypertension, and smoking have adverse effects in the foetus’s CV health [21]. 

Therefore, maternal risk prevention is the first step in the primordial prevention of hypertension development in children. During pregnancy, it is recommended to follow a low glycaemic index diet combined with a heart-healthy diet, engage in regular aerobic exercise if possible, and avoid smoking [21]. During childhood, maintaining a healthy weight, appropriate diet, and regular physical activity are crucial in preventing the development of hypertension [21]. Not only during the pregnancy, but the role of parental influence on the children’s health behaviour is important in maintaining a healthy lifestyle in a child since families share both genetic and environmental factors. Studies have shown similarities in child-family eating and exercise habits, BP readings, cholesterol levels, and body type with fat distribution [26].

Parents significantly impact their children’s health behaviour, influence their behaviour beyond adolescence, and a parent or adolescent’s definition of health can predict health-promoting behaviour, especially for girls [26]. According to social cognitive theory, parents are a major influence in the social environment and must be considered when implementing primordial or primary prevention models to reduce CV risk factors in children. Successful models of prevention include home and family interventions, which have led to more significant and sustained positive behaviour changes, especially in increased physical activity and greater self-efficacy for diet and exercise behaviour changes compared to school-based interventions [26].

In primordial prevention, national policies play an important role. Healthcare policymakers should prioritise ensuring access to healthy food, regular physical activity, high-quality healthcare, and health education. Various national measures can be implemented such as offering healthy diets and physical activity programs in schools, launching media campaigns to promote exercise and a low-sodium diet, and collaborating with food industries to reduce the sodium content in foods [21]. These strategies also have a positive effect on other cardio-metabolic conditions such as obesity, diabetes, and dyslipidaemias. Treating these conditions constitutes the primary prevention of hypertension, while preventing their development in the first place represents the primordial prevention of hypertension.

In the primary prevention of hypertension development, obesity plays a major role along with other aforementioned factors (genetics, intrauterine factors and perinatal conditions, diet, physical inactivity, sleep etc.). Obesity, coupled with an unhealthy diet and lack of physical exercise, has been a major contributor to nearly tripling the incidence of childhood hypertension in recent decades [27,28]. Obesity seems to affect hypertension development most significantly by various mechanisms such as insulin resistance, proinflammatory adipokines (leptin, resistin, for example) secretion, activation of the RAS system, salt retention, and resulting alterations in vascular endothelial function [10,28]. An increase in the prevalence of hypertension in children can therefore be directly linked to childhood obesity [27]. Similarly, during worldwide pandemics, a lack of regular physical activity and unhealthy diets have been shown to impact childhood obesity, consequently increasing the incidence of childhood hypertension [27]. Despite regular reports of the increasing prevalence of obesity and its consequences, among which hypertension is predominant, rates of childhood obesity are merely plateauing in some developed nations, and they appear to be rising in developing countries [27]. Given the impact of obesity in hypertension development along with inappropriate diet and a lack of physical activity, reducing weight by implementing the appropriate diet and exercise seems to be the most important public health strategy to reduce obesity-related childhood hypertension [27].

Nutritional intervention is globally acknowledged as the first-line treatment for individuals with increased cardiovascular risk, and it is especially crucial for children and adolescents. Current scientific evidence emphasises the importance of dietary patterns rather than individual nutrients or foods in preventing cardiovascular risk. In adults, DASH, or Dietary Approaches to Stop Hypertension, is a diet plan created by the National Institutes of Health to prevent and manage hypertension in adults. The DASH diet emphasises fruits, vegetables, whole grains, low-fat dairy products, and high-protein foods like nuts, beans, seeds, poultry, and fish. It restricts the intake of red meat, sugar, salt, and saturated fats, all of which contribute to hypertension [29]. However, in children, the main effects of the diet demonstrated the maintenance of normal growth in children with obesity/insulin resistance. The studies in paediatric hypertension are limited. Other dietary patterns might be appropriate in cardiovascular risk management such as the Mediterranean diet, plant based diet, Nordic diet, low-carb diet, ketogenic diet, Palaeolithic diet, and high protein diet [29]. Among these, the plant based diet (vegan, ovo-lacto-vegetarian) and Nordic diet (fish/shellfish, rapeseed oil/canola oil, low-fat dairy foods, nuts, legumes, berries, fruits, vegetables, whole-grain products) were shown to affect BP in children [29]. In adolescents at risk for hypertension, those with higher intakes of a combination of nutrients including potassium, calcium, magnesium, and vitamins (such as beta carotene, cholecalciferol, vitamin E, and all B vitamins) had lower BP, indicating an important role of micronutrient ingestion [30]. Dietary supplementation might be of even greater importance in children at an increased risk for hypertension such as low-birth-weight children. One study showed that iron supplements in infancy resulted in lower systolic BP later in life [31]. However, vitamin D supplementation was found to be ineffective in reducing systolic and diastolic BP in children and adolescents [32]. Further research is warranted to determine the effect of different micronutrients and dietary supplements in children at risk for hypertension development. With all diets, of course, it is necessary to ensure the intake of the necessary nutrients for the normal growth and development of children.

Increasing physical activity offers numerous benefits for children with mild hypertension or risk factors like obesity or a family history of hypertension. Regular exercise strengthens the heart, enhancing its efficiency and reducing arterial pressure. Additionally, exercise aids in weight reduction, which can lower BP and mitigate the risk of other hypertension-related conditions. Research indicates that incorporating 30 to 60 min of moderate aerobic activity into a child’s daily routine, while limiting sedentary activities like watching television or playing video games to less than two hours a day, can lower BP and decrease the need for antihypertensive medications. Specifically, compared to boys who engage in moderate to vigorous physical activity, those with a sedentary lifestyle may have an increased risk of high BP and high diastolic BP. Sedentary activity may be positively associated with systolic BP in both sexes, while moderate to vigorous physical activity may be negatively associated with diastolic BP in boys [33].

## 6. Secondary Prevention

Given the prevalence of elevated BP and hypertension in asymptomatic children, elevated BP should be regarded as a long-term health concern in paediatrics. The cornerstone of secondary preventive measures is the timely detection of elevated BP or hypertension, achieved through accurate BP measurement. In adults, this is routinely undertaken, however, in children not so often, despite their advantages (low cost, relatively quick, non-invasive). This is due to several challenges in children. Firstly, appropriately sized cuffs, which are not always available in all clinical settings, must be used. Additionally, interpreting the readings requires a reference to normal values based on age, height, and sex, necessitating the establishment of these values in specific populations and ensuring easy access to them [34]. Most widely accepted clinical recommendations differ (Table 1), however, they were found to significantly predict the risk of elevated BP in adulthood, though the 2017 American Academy of Pediatrics clinical practice guidelines yielded a higher hypertension prevalence [35].

Crying toddlers or agitated children might display falsely elevated BP [34] as is true for white coat hypertension, which according to some studies accounts for 32–46% of hypertension referrals [7]. Most commonly, oscillometric devices are used [36].

In children <3 years of age, high risk populations that should be screened for BP elevation include prematurity <32 week’s gestation, small for gestational age or low birth weight, history of umbilical artery line, congenital heart disease, recurrent urinary tract infections or abnormal urinalysis, known renal disease or urologic malformations, family history of renal disease, history of solid-organ transplant, malignancy or bone marrow transplant, treatment with medications known to increase BP, presence of other systemic illnesses associated with hypertension (neurofibromatosis, tuberous sclerosis, etc.), and evidence of elevated intracranial pressure [7]. Otherwise, for children aged 3 years and older, BP should be measured annually or at every routine healthcare visit [36]. More frequent monitoring is recommended for children who have obesity, are taking medications known to increase BP, have kidney disease, diabetes, or a history of congenital heart disease [36].

Detecting elevated BP or hypertension in children, whether primary or secondary, dictates further treatment. Its goal is to attain a BP level that decreases the risk of target organ damage during childhood and reduces the likelihood of hypertension and associated CVDs in adulthood. The presence of a secondary cause allows for targeted treatment [9]. Additionally, lifestyle modifications, already emphasised in primordial and primary prevention (appropriate diet, physical activity etc.) and sometimes complemented with pharmacological therapy, are advised. These measures are the cornerstone of treatment for primary hypertension [9]. Sometimes, lifestyle modifications fail to achieve a lowering of BP. In that case, pharmacologic therapy is indicated. Angiotensin convertase inhibitors (ACEi), angiotensin receptor blockers (ARBs), calcium channel blockers (CCBs), and diuretics are regarded as acceptable first-line antihypertensive agents according to current paediatric hypertension guidelines, whereas beta-blockers (BBs) are not recommended as first-line therapy. Studies have shown that ACEi are the most common antihypertensive agents used. ACEi and ARBs are especially appropriate for patients with chronic kidney disease or proteinuric conditions due to their nephroprotective and antiproteinuric effects. These are generally considered as a suitable choice for most children including those with primary hypertension [37]. Commonly used medications with their doses are presented in Table 3 [3,9].

## 7. Tertiary Prevention

Tertiary prevention focuses on disease complications. In childhood hypertension, these most commonly include short- and long-term complications, as presented in Table 4 [9].

To mitigate these complications, it is crucial to diagnose and subsequently manage hypertension early and more aggressively in children. In addition to lifestyle modifications, pharmacologic treatment is more frequently required when complications are present. Early intervention can significantly reduce the risk of both immediate and long-term complications associated with childhood hypertension [9].

The management of each child with elevated BP or hypertension should include early-stage investigations to detect possible complications. These investigations include heart and abdominal ultrasounds, intima-media thickness measurement, fundoscopic examination by an ophthalmologist, and pulse wave velocity measurement (a novel measure of arterial elasticity). Additional laboratory work includes assessing kidney function markers, lipids, markers of liver damage, and a 24-h urine sample for proteinuria as well as albumin/creatinine ratio in a morning void sample. For children with obesity, an oral glucose tolerance test with insulin levels should be conducted, along with body composition measurement, which should be followed with implemented lifestyle modifications. Several novel biomarkers are currently being studied as potential indicators of cardiovascular health and may be incorporated into cardiovascular risk stratification as more evidence becomes available in the future [38].

## 8. Quaternary Prevention

Quaternary prevention seeks to avoid unnecessary or harmful medical interventions including unwarranted examinations and pharmacotherapy.

Quaternary prevention in hypertension management in children includes [9,39]:

▪ Avoiding overdiagnosis: Ensuring that hypertension diagnosis is accurate and not based on isolated high readings or non-standardised measurements.
▪ Judicious use of medications: Preventing unnecessary pharmacotherapy by first exploring and implementing lifestyle modifications such as diet and exercise before resorting to medication.
▪ Minimising unnecessary tests: Avoiding excessive diagnostic tests that do not contribute to the management or outcome of the condition, reducing the physical and psychological burden on the child.
▪ Education and communication: Educating families about the importance of lifestyle changes and the potential risks of unnecessary treatments, fostering informed decision making.
▪ Monitoring and follow-up: Regularly reviewing the necessity and effectiveness of any prescribed treatments, ensuring that interventions remain appropriate and are not continued longer than necessary.
▪ Preventing medicalisation: Focusing on holistic approaches that consider the child’s overall well-being rather than solely treating the medical condition, thereby avoiding the medicalisation of normal variations in BP.

## 9. Conclusions

Identifying, examining, preventing, and treating children with high-risk BP is crucial for both clinical and public health benefits. This step is essential in reducing the excessive burden of CVDs. In children, primordial and preventive measures should be undertaken with an emphasis on family lifestyle modifications. High-risk BP should be detected regularly with appropriate BP measurements as it is an inexpensive, relatively quick, and non-invasive method for screening children for hypertension. Timely treatment allows for the early prevention of complications and subsequent CVD development, the number one cause of morbidity and mortality worldwide.

## Figures and Tables

**Table 1:** Definitions of elevated blood pressure and hypertension based on the 2016 European Society of Hypertension guidelines, the 2017 American Academy of Pediatrics clinical practice guidelines and Hypertension Canada’s 20202 guidelines.

	2016 European Society of Hypertension Guidelines		2017 American Academy of Pediatrics Clinical Practice Guidelines		Hypertension Canada’s 2020 Guidelines	
	<16 Years	≥16 Years	<13 Years	≥13 Years	6–11 Years	12–17 Years
Normal BP	<90th percentile	<130/85 mmHg	<90th percentile	<120/<80 mmHg	<95th percentile or <120/80 mmHg	<95th percentile or <130/85 mmHg
Elevated BP	≥90th to <95th percentile	130–139/85–89 mmHg	≥90th to < 95th percentile or 120–129/<80 mmHg (whichever is lower)	120/<80 to 129/<80 mmHg	Not defined	Not defined
Stage 1 hypertension	≥95th to <99th percentile + 5 mmHg	140–159/90–99 mmHg	≥95th percentile to <95th percentile + 12 mmHg, or 130/80 to 139/89 mmHg (whichever is lower)	130/80 to 139/89 mmHg	95th–95th percentile + 12 mm Hg	
Stage 2 hypertension	≥99th percentile + 5 mmHg	160–179/100–109 mmHg	≥95th percentile + 12 mmHg, or ≥140/90 mmHg (whichever is lower)	≥140/90 mmHg	>95th percentile + 12 mm Hg	

**Table 2:** Classification of preventive measures.

Type/Level of Prevention	Aim of Measures
Primordial	Control of single risk factors in a normal range (healthy lifestyle, body weight control)
Primary	Prevention of disease (education focused on maintaining or restoring a healthy lifestyle)
Secondary (early)	Detection of the disease in its early, asymptomatic stage (screening, medical treatment)
Tertiary (late)	Preventing disease complications
Quaternary	Preventing unnecessary or harmful medical activities (preventing unnecessary examinations, pharmacotherapy)

**Table 3:** Antihypertensive drugs for the treatment of hypertension in children and adolescents.

Drug Category	Drug	Initial Daily Oral Dose	Maximum Daily Oral Dose	Dosing Interval	Recommended Age
ACE inhibitors	Benazepril	0.2 mg/kg max: 10 mg	0.6 mg/kg max: 40 mg	Once/day	≥6 years
	Captopril	0.05 mg/kg/dose (max: 40 mg) 0.5 mg/kg/dose	6 mg/kg 6 mg/kg	Four times/day Three times/day	Babies Children
	Enalapril	0.08 mg/kg max: 5 mg	0.6 mg/kg	Once or twice daily	≥1 month
	Fosinopril	0.1–0.6 mg/kg max: 5 mg	40 mg	Once/day	≥6 years
	Lisinopril	0.07 mg/kg max: 5 mg	0.6 mg/kg max: 40 mg/day	Once/day	≥6 years
	Ramipril	1.6 mg/m^2^	6 mg/m^2^	Once/day	
Angiotensin receptor blockers	Candesartan	0.16–0.5 mg/kg	<50 kg: 16 mg >50 kg: 32 mg	Once/day	≥1 year
	Irbesartan	75 mg 150 mg	150 mg 300 mg	Once/day	6–12 years ≥13 years
	Losartan	0.7 mg/kg max: 50 mg	1.4 mg/kg max: 100 mg	Once/day	≥6 years
	Olmesartan	<35 kg: 10 mg >35 mg: 20 mg	20 mg 40 mg	Once/day	≥6 years
	Valsartan	0.4 mg/kg	40–80 mg	Once/day	≥6 years
Beta-blockers	Atenolol	0.5–1 mg/kg	2 mg/kg max: 100 mg	Once or twice daily	
	Metoprolol	0.5–1 mg/kg	2 mg/kg	Once or twice daily	
	Propranolol	1 mg/kg	4 mg/kg max: 640 mg	Twice or three times daily	
Diuretics	Chlorthalidone	0.3 mg/kg	2 mg/kg max: 50 mg	Once/day	Children
	Chlorothiazide	10 mg/kg	20 mg/kg max: 375 mg	Once or twice daily	Children
	Hydrochlorothiazide	1 mg/kg	2 mg/kg max: 37.5 mg	Once or twice daily	Children
	Amiloride	0.4–0.6 mg/kg	20 mg	Once/day	
	Spironolactone	1 mg/kg	3.3 mg/kg max: 100 mg	Once or twice daily	
	Furosemide	0.5–2 mg/kg	6 mg/kg	Once or twice daily	
	Eplerenone	25 mg	100 mg	Once or twice daily	
	Triamterene	1–2 mg/kg	3–4 mg/kg		
Calcium channel blockers	Amlodipine	0.1 mg/kg 0.3 mg/kg	5 mg 10 mg	Once/day	1–5 years ≥6 years
	Felodipine	2.5 mg	10 mg	Once/day	≥6 years
	Isradipine	0.05–0.1 mg/kg	0.6 mg/kg max: 10 mg	Twice or three times daily	Children
	Nifedipine	0.25–0.5 mg/kg	3 mg/kg max: 120 mg	Once or twice daily	
Alpha- and beta blockers	Labetalol	1–3 mg/kg	10–12 mg/kg max: 1200 mg	Once/day	
Central alpha agonists	Clonidine	0.2 mg/kg	2.4 mg/kg	Twice/day	
Peripheral alpha blockers	Doxazosin	1 mg	4 mg	Once/day	
	Prazosin	0.05–0.1 mg/kg	0.5 mg/kg	Three times/day	
Vasodilators	Hydralazine	0.75 mg/kg	7.5 mg/kg max: 200 mg	Four times/day	
	Minoxidil	0.2 mg/kg	50–100 mg	Once to three times daily	

**Table 4:** Short- and long-term complications of hypertension in children.

Short-Term Complications	Long-Term Complications
Left ventricular hypertrophy	Persistent hypertension
Vascular damage (endothelial dysfunction)	Cardiovascular disease
Kidney damage (proteinuria, chronic kidney disease)	Metabolic syndrome
Eye damage (hypertensive retinopathy)	Impaired cognitive function
Cerebrovascular effects (very rare)	Psychosocial impact
